# The Impact of Mevastatin on HCV Replication and Autophagy of Non-Transformed HCV Replicon Hepatocytes Is Influenced by the Extracellular Lipid Uptake

**DOI:** 10.3389/fphar.2019.00718

**Published:** 2019-06-26

**Authors:** Tiziana Vescovo, Giulia Refolo, Matteo Ciancio Manuelli, Giuseppe Tisone, Mauro Piacentini, Gian Maria Fimia

**Affiliations:** ^1^Department of Epidemiology, Preclinical Research and Advanced Diagnostics, National Institute for Infectious Diseases Lazzaro Spallanzani-IRCCS, Rome, Italy; ^2^Liver Unit, Polyclinic Tor Vergata Foundation, University of Rome Tor Vergata, Rome, Italy; ^3^Department of Biology, University of Rome Tor Vergata, Rome, Italy; ^4^Department of Biological and Environmental Sciences and Technologies (DiSTeBA), University of Salento, Lecce, Italy

**Keywords:** hepatitis C virus, cholesterol, autophagy, RNA replication, lipid uptake

## Abstract

Statins efficiently inhibit cholesterol synthesis by blocking 3-hydroxy-3-methylglutaryl (HMG)-CoA reductase in the mevalonate pathway. However, the effect of statins on intracellular cholesterol is partially counterbalanced by a consequent increased uptake of extracellular lipid sources. Hepatitis C virus (HCV) infection induces intracellular accumulation of cholesterol by promoting both new synthesis and uptake of circulating lipoproteins, which is required for HCV replication and release. Hepatocytes respond to the increase in intracellular cholesterol levels by inducing lipophagy, a selective type of autophagy mediating the degradation of lipid deposits within lysosomes. In a cellular system of HCV replication based on HuH7 hepatoma cells, statin treatment was shown to be sufficient to decrease intracellular cholesterol, which is accompanied by reduced HCV replication and decreased lipophagy, and has no apparent impact on endocytosis-mediated cholesterol uptake. To understand whether these results were influenced by an altered response of cholesterol influx in hepatoma cells, we analyzed the effect of statins in non-transformed murine hepatocytes (MMHD3) harboring subgenomic HCV replicons. Notably, we found that total amount of cholesterol is increased in MMHD3 cells upon mevastatin treatment, which is associated with increased HCV replication and lipophagy. Conversely, mevastatin is able to reduce cholesterol amounts only when cells are grown in the presence of delipidated serum to prevent extracellular lipid uptake. Under this condition, HCV replication is reduced and autophagy flux is severely impaired. Altogether, these results indicate that both *de novo* synthesis and extracellular uptake have to be targeted in non-transformed hepatocytes in order to decrease intracellular cholesterol levels and consequently limit HCV replication.

## Introduction

Hepatitis C virus (HCV) dysregulates lipid metabolism to accomplish several steps of its life cycle (Paul et al., 2014; Strating and van Kuppeveld, 2017). HCV associates with lipoproteins to circulate in the bloodstream, to enter into and to be released from target cells. It induces the formation of a membranous web in close proximity to the endoplasmic reticulum (ER) where its replication complex is assembled. In this regard, a crucial role is played by cholesterol, whose intracellular levels are increased during HCV infection by stimulating both new synthesis and uptake from extracellular milieu (Ye, 2007; Bassendine et al., 2011; Grassi et al., 2016; Lavie and Dubuisson, 2017).

Cholesterol is synthesized from acetyl CoA through the mevalonate pathway at the ER, mainly in the liver and intestines (Edwards and Ericsson, 1999). The conversion of 3-hydroxy-3-methylglutaryl (HMG)-CoA in mevalonate by HMG-CoA reductase is the rate-limiting step of the cholesterol biogenesis. Once synthesized, cholesterol is rapidly targeted to biological membranes or distributed to intracellular deposits, such as lipid droplets, where it undergoes fatty acylation to generate cholesterol esters. An alternative source of cholesterol is represented by the uptake of low-density lipoproteins (LDLs) from serum through LDL receptor-mediated endocytosis (Ikonen, 2008; Arab et al., 2018). Cholesteryl esters that enter the cells through LDL are hydrolyzed in the lysosomes to provide unesterified cholesterol for cellular needs.

Statins inhibit cholesterol synthesis by blocking mevalonate production *via* inhibition of HMG-CoA reductase (Davies et al., 2016). Statin-treated cells respond to the reduction in the synthesis rate by increasing the number of LDL receptors on cell surface to ensure the uptake of cholesterol from serum (Davies et al., 2016).

Studies based on HCV RNA replicon or HCV-infected cells showed that HCV stimulates cholesterol synthesis, and statins markedly reduce RNA replication and viral particle infection (Ye et al., 2003; Kapadia and Chisari, 2005; Kapadia et al., 2007). This inhibition is caused by the reduced availability of cholesterol for membranous web formation and for viral lipoprotein assembly, as well as by the impairment of post-translational modifications of host proteins involved HCV replication (e.g., FBL2 geranylgeranylation), which depends on mevalonate pathway intermediates (Wang et al., 2005).

In addition to increased cholesterol synthesis, HCV is also able to induce cholesterol uptake from serum *via* upregulation of LDL receptor expression (Syed et al., 2014; Zhang et al., 2017). Blocking this pathway affects HCV replication, similar to what was observed with statins, suggesting that both sources of cholesterol are important in the viral cycle. It has been recently reported that inhibition of cholesterol transport through the endosomal–lysosomal pathway impairs the formation of the membranous web, where RNA replication occurs (Stoeck et al., 2017).

Autophagy is a lysosome-mediated catabolic process that ensures cellular integrity and homeostasis (Mizushima and Komatsu, 2011) in response to several stress stimuli, such as nutrient deprivation, accumulation of harmful substrates, or infection (Kroemer, 2015). In this process, double-membrane vesicles, called autophagosomes, engulf portions of cytoplasm and transport them to lysosomes for degradation (Antonioli et al., 2017). Different types of autophagy selective for specific cargos have been described (Khaminets et al., 2016). Among them, lipophagy controls intracellular lipid homeostasis, allowing the hydrolysis of intracellular triglycerides and cholesterol esters stored in lipid droplets (Liu and Czaja, 2013;Vescovo et al., 2014; Madrigal-Matute and Cuervo, 2016). Accordingly, lipophagy is induced in hepatocytes treated with statins to compensate for cholesterol synthesis decrease (Wang et al., 2015).

We have previously reported that HCV-infected HuH7 cells have high rate of lipophagy, whose inhibition results in a significant accumulation of cholesterol (Vescovo et al., 2012). Statin treatment in these cells is sufficient to reduce intracellular cholesterol levels, which is accompanied by a reduction of both lipophagy and HCV replication. However, a limitation of this study was the use of hepatoma cells, which may have alterations in the regulation of cholesterol homeostasis. Here, we analyzed how statins impact HCV replication, cholesterol levels, and autophagy in the non-transformed mouse hepatocytes MMHD3. These cells were previously reported to support replication of a JFH1-derived subgenomic replicon (HCV Rep), although species restriction impedes both viral entry and production of new viral particles (Uprichard et al., 2006).

## Materials and Methods

### Cell Culture

HuH7 HCV-Rep is a human hepatoma cell line harboring the HCV genotype 1b (Con1) subgenomic replicon carrying a neomycin resistance gene (Vescovo et al., 2012). Immortalized Met Mouse Hepatocytes D3 (MMHD3) HCV-Rep is a mouse cell line harboring an HCV subgenomic replicon derived from JFH1 genotype 2a carrying a neomycin resistance gene (Uprichard et al., 2006). Cells were cultured at 37°C in 5% CO_2_, and HuH7 HCV-Rep and HEK293T were grown in Dulbecco’s Modified Eagle’s medium (DMEM) (Sigma-Aldrich, St Louis, MO) while MMHD3 HCV-Rep cells were grown in Roswell Park Memorial Institute (RPMI) 1640 medium with the addition of 30 ng/ml insulin-like growth factor II (IGFII), 50 ng/ml epidermal growth factor (EGF) (Peprotech), and 10 µg/ml insulin (Roche). DMEM and RPMI were supplemented with 10% fetal bovine serum (FBS) (Gibco), 100 U/mL penicillin, 100 g/mL streptomycin, and 2 mM L-glutamine (Sigma-Aldrich, St Louis, MO). To maintain HCV replication, culture medium of HuH7 and MMHD3 HCV replicon cells was added with 500 μg/ml and 250 μg/ml G418 (Sigma-Aldrich, St Louis, MO), respectively. G418 was removed in the course of the experiments.

To inhibit cellular cholesterol uptake, cells were cultured in RPMI supplemented with 10% delipidated FBS (Pan-Biotech, Aidenbach, Germany). To inhibit cholesterol synthesis, cells were incubated in complete or delipidated FBS medium in the presence of 5 µM mevastatin (Sigma-Aldrich, St Louis, MO) for 72 h before the assay.

To block lysosomal activity, cells were incubated in complete or delipidated FBS medium in the presence of 5 nM Bafilomycin A1 (Sigma-Aldrich, St Louis, MO) for 4 and 8 h before the assay. RFP-LC3 and RFP-GFP-LC3 retroviral reporters and methods for retrovirus production were previously described (Antonioli et al., 2014).

### Lipid Staining and Quantification

Cells were fixed in 4% paraformaldehyde in phosphate-buffered saline (PBS) for 20 min, washed thoroughly with PBS, and incubated with filipin (50 μg/ml;Sigma-Aldrich, St Louis, MO) or Bodipy 493/503 (2 μg/ml; Invitrogen, Carlsbad, CA) for 1 h at room temperature.

For luminometric quantification, 6 × 10^5^ cells were trypsinized, washed thoroughly with PBS, and fixed with 4% paraformaldehyde in PBS for 20 min at room temperature. Cells were then washed twice with PBS, split in two tubes, and incubated with fluorescence probes indicated above. Cells were finally washed thoroughly with PBS and distributed in flat-bottom 96-well plates (OptiPlate-96 F; Perkin Elmer, Waltham, MA). Fluorescence was measured using a Victor 3 1420 multilabel plate reader (Perkin Elmer, Waltham, MA) and the following filter pairs: 485/535 nm for Bodipy and 355/460 nm for 4′,6-diamidino-2-phenylindole and filipin. Fluorescence intensities were normalized with respect to 4′,6-diamidino-2-phenylindole. Assays were performed in triplicate.

For cholesterol quantitation, a Cholesterol Quantitation Kit (MAK043, Sigma-Aldrich, St Louis, MO) was used, and 1 × 10^6^ cells were processed according to the manufacturer’s recommendations. Cholesterol measurement was normalized with respect to the quantity of DNA extracted with Trizol reagent (Invitrogen, Carlsbad, CA). Assays were performed in triplicate.

### Antibodies and Immunoblotting Analysis

The primary antibodies used in this study were rabbit anti-LC3 (Cell Signaling Technology, Inc., Danvers, MA, for Western blot analysis) and α-glyceraldehyde-3-phosphate dehydrogenase (Calbiochem, Merck, Darmstadt, Germany). Cells were lysed in Cell Lytic (Sigma-Aldrich, St Louis, MO) complemented with protease and phosphatase inhibitors (Protease inhibitor cocktail plus 5 mM sodium fluoride, 0.5 mM sodium orthovanadate, 1 mM sodium molibdate, and 0.5 mM phenylmethylsulfonyl fluoride; Sigma-Aldrich, St Louis, MO). Ten micrograms of protein extracts was separated on 13.5% sodium dodecyl sulfate-polyacrylamide gel electrophoresis gel and electroblotted onto polyvinylidene difluoride (Millipore, Billerica, MA) membranes. Blots were incubated with primary antibodies in 5% nonfat dry milk in PBS plus 0.1% Tween 20 overnight at 4°C. Detection was achieved using horseradish peroxidase–conjugated secondary antibody (Jackson ImmunoResearch Laboratories, West Grove, PA), visualized with ECL (Millipore, Billerica, MA), and chemiluminescent signals were analyzed using a Chemidoc Touch Imaging System (Bio-Rad, Hercules, CA). All samples were run in triplicate.

### Lentivirus Generation and Infection

Lentiviral infections were performed as previously described (Di Rienzo et al., 2019). Briefly, HEK 293T cells were cotransfected with 10 μg of lentiviral vector targeting Beclin 1 messenger ribonucleic acid (mRNA) (Sigma-Aldrich, St Louis, MO), 2.5 μg of pVSV-G plasmid, and 7.5 μg of psPAX2 plasmid using the calcium phosphate method. After 48 h, lentiviral particles were recovered from the supernatants by ultracentrifugation at 19,800 RPM on SW28 rotor for 2 h and resuspended in PBS (500 μl for 20 ml of culture media). Cells were infected with 40 μl of viral suspension supplemented with 4 μg/ml polybrene (Sigma-Aldrich, St Louis, MO) overnight. Transduced cells were selected for puromycin resistance (250 μg/ml; Sigma-Aldrich, St Louis, MO).

### Real-Time Polymerase Chain Reaction

Analysis of HCV RNA was performed as previously described (Refolo et al., 2019). Briefly, RNA was extracted with Trizol reagent (Invitrogen, Carlsbad, CA). Complementary DNA synthesis was generated from 2 μg of RNA using the reverse transcription kit (Promega, Madison, WI) according to the manufacturer’s recommendations. Real-time polymerase chain reactions (PCRs) were performed with the Corbett Research Rotor Gene 6000 analyzer using the Maxima SYBR Green/ROX qPCR Master Mix (Thermo Scientific, Waltham, MA) according to the manufacturer’s instructions. Two point five microliters of 1:5 complementary deoxyribonucleic acid (cDNA) was used as template and cycling parameters were 95°C for 10 min, followed by 40 cycles of 95°C for 15 s, 60°C for 30 s, and 72°C for 30 s.

Levels of RNAs were normalized to the L34 level using the equation 2^−ΔCt^.

Primer sets for all amplicons were designed using the Primer-Express 1.0 software system (Roche, Basel, CH).

Replicon genotype 1b forward: 5’-TACTCCCAACAGACGCGAGG-3’ 

Replicon genotype 1b reverse: 5’-GCAGGTCGCCAGGAAAGATT-3’ 

Replicon genotype 2a forward: 5’-TCTGCGGAACCGGTGAGTA-3’ 

Replicon genotype 2a reverse: 5’-TCAGGCAGTACCACAAGGC-3’ 

L34 human forward: 5’-GTCCCGAACCCCTGGTAATAG-3’ 

L34 human reverse: 5’-GGCCCTGCTGACATGTTTCTT-3’ 

L34 mouse forward: 5’-GGTTGGGAAAGCACCTAAA-3’ 

L34 mouse reverse: 5’-GACGTGCTTCTGTGTCTTAG-3’ 

Beclin 1 mouse forward: 5’-GGCCAATAAGATGGGTCTGA-3’ 

Beclin 1 mouse reverse: 5’-GCTGCACACAGTCCAGAAAA-3’

### Statistical Analysis

Statistical analysis was performed using unpaired, two-tailed Student’s *t* test (Excel software). Values are shown as mean ± standard deviation of at least three independent experiments. *P* values <0.05 were marked by *. Densitometric analysis of immunoblots was performed using the Adobe Photoshop software.

## Results

**Figure 1:**
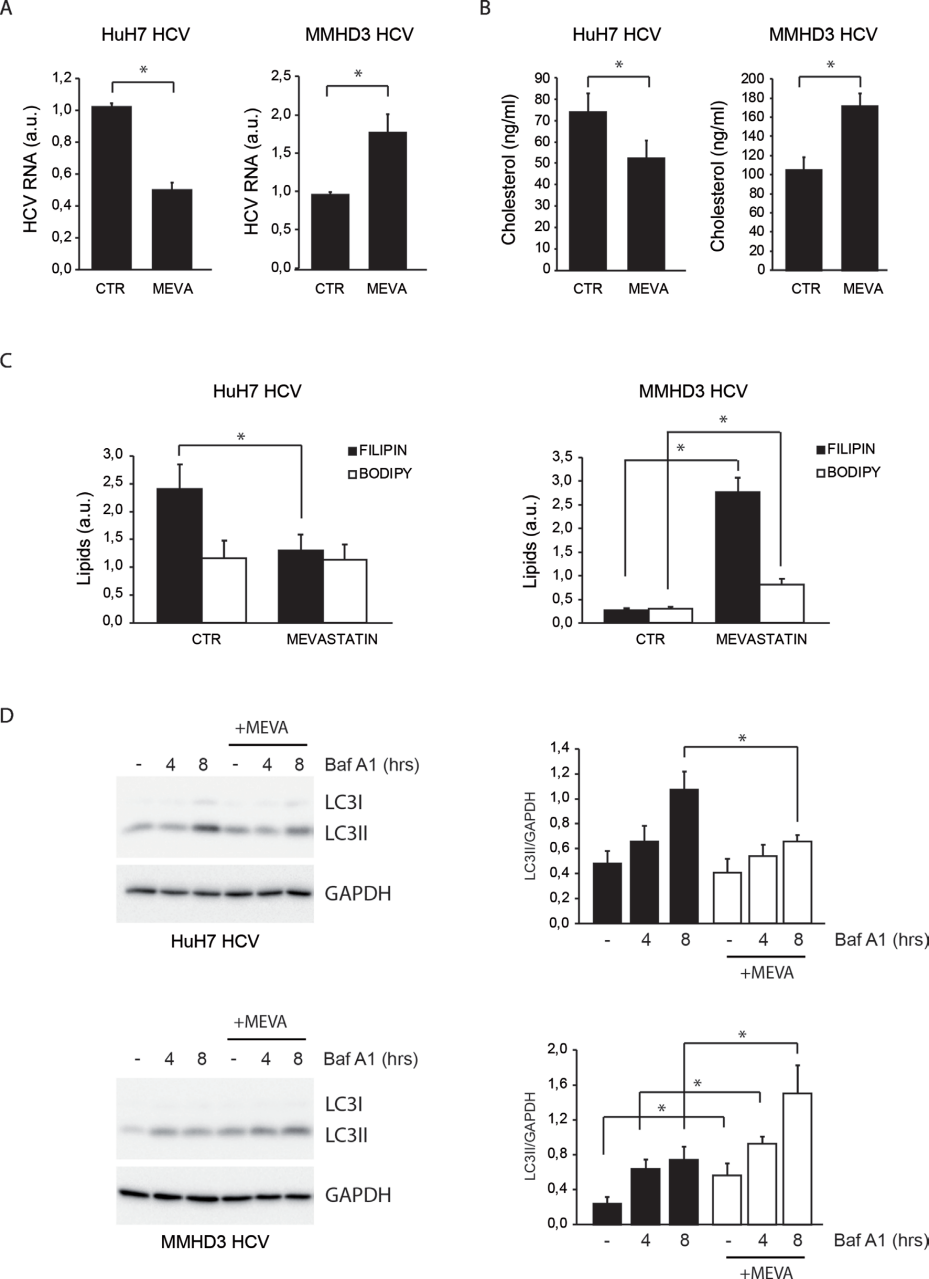
Comparison of the response to mevastatin treatment of HuH7 and MMHD3 hepatitis C virus (HCV) Rep cell lines in terms of HCV replication, cholesterol, and autophagy levels. **(A)** HuH7 HCV-Rep (left panel) and MMHD3 HCV-Rep (right panel) cells were treated with mevastatin for 3 days and HCV replicon levels were analyzed by real-time polymerase chain reaction (PCR). A.U.: Arbitrary units. **(B)** HuH7 HCV-Rep (left panel) and MMHD3 HCV-Rep (right panel) cells were treated with mevastatin for 3 days and intracellular cholesterol content was measured using cholesterol quantitation kit (Sigma-Aldrich). **(C)** HuH7 HCV-Rep (left panel) and MMHD3 HCV-Rep (right panel) cells were treated with mevastatin for 3 days and intracellular lipid content was analyzed by staining cells with filipin (cholesterol) and Bodipy 493/503 (neutral lipids) and measuring fluorescence intensity using a fluorimeter. **(D)** HuH7 HCV-Rep (top panel) and MMHD3 HCV-Rep (bottom panel) cells were incubated with mevastatin (MEVA) for 3 days and autophagic flux was analyzed by treating, or not, cells with 5 nM Bafilomycin A1 (Baf A1) for 4 or 8 h. Protein extracts were prepared and subjected to immunoblotting to determine LC3 protein levels. Glyceraldehyde 3-phospate dehydrogenase (GAPDH) was used as a protein loading control. Accompanying graphs show LC3II values normalized to GAPDH levels from three independent experiments. *P < 0.05.

MMHD3 HCV Rep was treated with mevastatin for 3 days and HCV replication was evaluated by real-time PCR. HuH7 HCV replicon cells were also included in this study as a positive control. Unexpectedly, while mevastatin impairs HCV replication in HuH7 HCV Rep cells, a significant increase of HCV RNA levels was observed in MMHD3 Rep cells (**Figure 1A**).

To elucidate whether the discordant effect of mevastatin on HCV replication was due to a different impact on the intracellular cholesterol amount, cholesterol levels were analyzed upon mevastatin incubation by both enzymatic assays and filipin staining (**Figures 1B and C** and **S1A**). Results show that mevastatin treatment reduces cholesterol levels in HuH7 Rep cells, while it augments them in MMHD3 Rep cells. Neutral lipid levels were also analyzed by Bodipy 493/503 staining, showing no changes in HuH7 Rep cells and a significant increase in MMHD3 Rep cells (**Figures 1C** and **S1A**).

We previously described that high levels of a lipid-selective autophagy are present in HuH7 Rep cells as a response to cholesterol accumulation triggered by HCV replication, which can be diminished by statin treatment (Vescovo et al., 2012). In light of the different response of MMHD3 Rep cells to mevastatin, we analyzed autophagy flux in these experimental conditions by measuring the autophagic marker LC3-II in the presence or absence of the lysosomal inhibitor Bafilomycin A1. As shown in **Figure 1D**, mevastatin treatment decreases autophagy levels in HuH7 Rep cells, while this was further increased in MMHD3 Rep cells in accordance with increased levels of cholesterol. We also confirmed that, in MMHD3 Rep cells, autophagosomes largely localized with cholesterol deposits, as shown by LC3 and filipin colocalization (**Figure S1B**). Altogether, these results suggests that, in non-transformed HCV replicating hepatocytes, statins cause an increase rather than a decrease of total cholesterol levels, which is accompanied by increased levels of autophagy.

**Figure 2:**
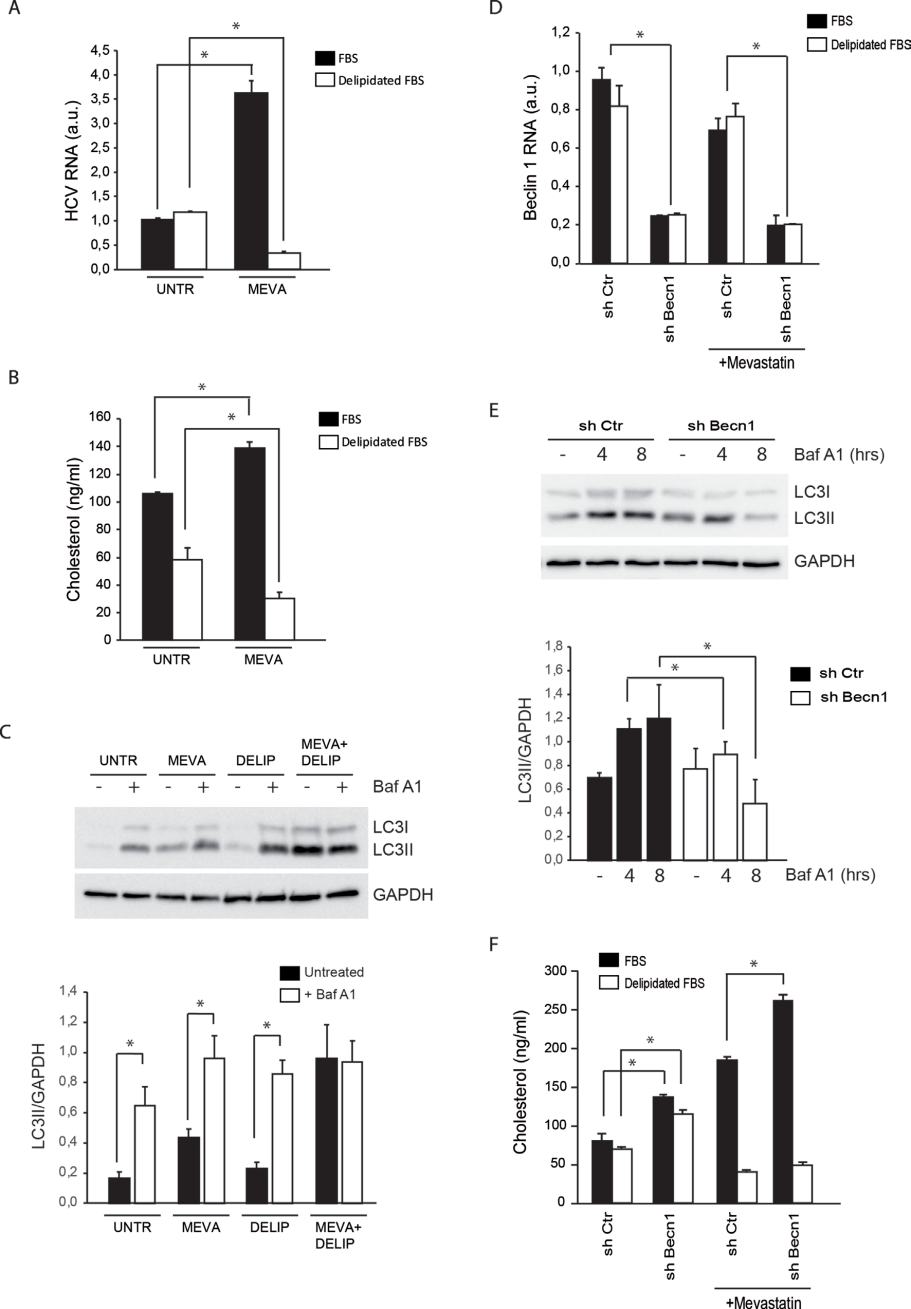
Analysis of HCV replication, cholesterol, and autophagy levels in MMHD3 HCV Rep cells treated with mevastatin and delipidated serum. **(A)** MMHD3 HCV-Rep cells were treated with mevastatin for 3 days in the presence of normal or delipidated fetal bovine serum (FBS). HCV replicon levels were analyzed by real-time polymerase chain reaction (PCR). **(B)** MMHD3 HCV-Rep cells were treated with mevastatin for 3 days in the presence of normal or delipidated FBS. Intracellular cholesterol content was measured using cholesterol quantitation kit (Sigma-Aldrich). **(C)** In MMHD3 HCV-Rep cells treated with mevastatin for 3 days in the presence of normal or delipidated FBS, autophagy flux was measured by treating, or not, cells with 5 nM of Bafilomycin A1 (Baf A1) for 8 h. Protein extracts were subjected to immunoblotting to determine LC3 protein levels. Glyceraldehyde 3-phospate dehydrogenase (GAPDH) was used as a protein loading control. The accompanying graph shows LC3II values normalized to GAPDH levels from three independent experiments. *P < 0.05. **(D)** MMHD3 HCV-Rep cells were infected with a sh-Control (Ctr) or a sh-Beclin 1 (Becn1) lentivirus and Beclin 1 levels were analyzed by real-time PCR. **(E)** Autophagy flux in Beclin 1-silenced cells were analyzed by immunoblotting as described in **(C)**. **(F)** Intracellular cholesterol content was measured in Beclin 1-silenced cells cultured in delipidated FBS and treated, or not, with mevastatin for 3 days using a cholesterol quantitation kit (Sigma-Aldrich). *P < 0.05.

Statin treatments are known to stimulate the extracellular uptake of lipoproteins as a cellular attempt to compensate for the cholesterol synthesis inhibition (Davies et al., 2016). The increase of neutral lipids associated with that of cholesterol suggests that extracellular lipid uptake is potentiated in MMHD3 Rep cells upon mevastatin incubation. To elucidate the role of extracellular cholesterol in the response of MMHD3 Rep cells, mevastatin treatments were repeated in cells cultured in medium containing delipidated serum. As shown in **Figure 2A**, HCV RNA levels were efficiently reduced by mevastatin when extracellular sources of cholesterol were also diminished. In parallel, cholesterol levels were analyzed by both enzymatic assays and filipin staining, showing that mevastatin is able to reduce cholesterol levels in MMHD3 Rep cells when cultured with delipidated serum (**Figures 2B** and **S1C**). Of note, lipid-deprived medium alone results in lower intracellular cholesterol levels; however, this decrease is not sufficient to impair HCV replication.

Then, we analyzed the autophagy flux in MMHD3 Rep cells cultured in serum-delipidated medium and mevastatin, either individually or in combination, and in the presence or absence of the lysosome inhibitor Bafilomycin A1 (**Figure 2C**). As expected, elimination of extracellular lipid sources result in autophagy induction, with nutrient starvation being a well-known inducer of autophagy. Interestingly, when cholesterol synthesis is also inhibited by mevastatin, we observed a block of LC3II degradation (autophagy flux) rather than a decrease of LC3II lipidation (autophagy induction), suggesting that, when total cholesterol becomes limiting, autophagosome–lysosome fusion or lysosomal degradative activity is affected. The impairment of autophagy flux in cholesterol-deficient conditions was also confirmed by using the tandem fluorescent-tagged LC3 (mRFP-EGFP-LC3, **Figure S1D**).

We further explored the role of autophagy in the control of cholesterol levels in MMHD3 Rep cells by inhibiting the process through downregulating the expression of the autophagy regulator Beclin 1 (**Figure 2D and E**). Beclin 1 silencing results in an accumulation of cholesterol in cells cultured either in complete or in delipidated serum. However, when Beclin 1-silenced cells were incubated with mevastatin, cholesterol accumulation was reduced only if extracellular sources of cholesterol were depleted, while a further accumulation was observed in cells incubated in complete medium. These results confirmed that when intracellular sources of cholesterol (new synthesis and autophagic degradation) are both inhibited, the uptake of extracellular cholesterol is significantly increased (**Figure 2F**).

## Discussion

HCV replication relies on the formation of the membranous web, a massive rearrangement of ER-associated membranes highly enriched in cholesterol (Paul et al., 2014). HCV promotes intracellular accumulation of cholesterol both by inducing new synthesis through the mevalonate pathway and by stimulating extracellular lipoprotein uptake (Bassendine et al., 2011; Strating and van Kuppeveld, 2017). The contribution of different sources of cholesterol to sustain viral replication remains partially characterized. Statins are powerful inhibitors of cholesterol synthesis. However, their effect on the total levels of intracellular cholesterol is attenuated by the ability of hepatocytes to upregulate the uptake of extracellular cholesterol by inducing LDL receptor expression (Davies et al., 2016).

Previous studies showed that inhibition of cholesterol synthesis by statins is sufficient to significantly lower total cholesterol levels in HuH7 HCV replicon cells, which is associated with reduced HCV replication (Ye, 2007; Vescovo et al., 2012). However, this observation also suggests that the compensatory increase of extracellular cholesterol uptake triggered by statins may be somehow impaired in HuH7 cells, raising the question if these cells properly reflect what occurs in normal hepatocytes.

Here, we reported that, in non-transformed HCV replicon hepatocytes, the impact of statins on cholesterol levels is profoundly influenced by the availability of extracellular lipid sources. Indeed, treatment of immortalized murine hepatocytes with mevastatin is not able to decrease intracellular cholesterol levels unless these cells are cultured in the presence of delipidated serum. In line with this observation, mevastatin alone causes an increase rather than a decrease of HCV replication, while it shows antiviral properties only when combined with the delipidated serum.

Statins were described to inhibit HCV replication not only by reducing cholesterol levels but also by inhibiting prenylation of host factors required for HCV replication (Ye et al., 2003; Kapadia and Chisari, 2005). Since mevastatin treatments alone do not impair HCV replication in immortalized hepatocytes, it remains to be assessed at which extent protein prenylation is inhibited. In this regard, it has to be noted that the maximum concentration of mevastatin at which immortalized hepatocytes can be treated is 5 µM, which is lower than what is commonly used in HuH7, since higher doses induce massive cell death (data not shown).

We previously reported that lipophagy, a lipid-selective form of autophagy, is induced by HCV infection in HuH7 cells, which is required to prevent excessive accumulation of intracellular cholesterol, and decreases when cells are treated with statins. As in the case of cholesterol levels and HCV replication, we observed that the effect of statins on autophagy in non-transformed HCV replicon hepatocytes is dependent on the presence of extracellular sources of lipids. At variance with HuH7 replicon cells, autophagy is increased by statin treatment. Interestingly, we observed that delipidated serum induces autophagy flux, probably as a response to nutrient starvation, while a dramatic inhibition of LC3II degradation was observed when combined to mevastatin, suggesting that profound cholesterol depletion may affect autophagosome degradation, as also described in breast cancer cells (Lin et al., 2017).

The impact of autophagy inhibition on cholesterol levels is also determined by extracellular lipid availability. An increase of cholesterol levels was observed when autophagy was inhibited by downregulating Beclin 1 expression in non-transformed hepatocytes. However, also in this case, mevastatin is able to reduce cholesterol levels only if Beclin 1-silenced cells are cultured in the presence of delipidated serum, while a further increase was observed when extracellular lipid sources were available.

Although our observations need to be validated using more physiological cellular models, such as HCV-infected primary hepatocytes, they highlighted alterations in transformed hepatocytes with respect to lipid metabolism that may have limited our understanding of the effect of inhibition of cholesterol synthesis in the context of HCV infection. Non-transformed HCV replicon hepatocytes may therefore represent a more physiological cell system to elucidate how lipid metabolism is altered by HCV, which is a key aspect of HCV-induced pathogenesis (Patel and Harrison, 2012;Vescovo et al., 2016).

## Author Contributions

TV performed most experiments with crucial help from GR (immunoblotting) and MM (cholesterol assays). GT, MP, and GF conceived and designed the research. TV and GF wrote the manuscript. All authors discussed the results and commented on the manuscript.

## Funding

This work was supported by grants from AIRC (IG2015 no. 17404 to GF and IG2018 to MP), the Italian Ministry of Health (Ricerca Corrente to MP and GF, and Ricerca Finalizzata GR-2013-02359524 to TV), and the Italian Ministry of University and Research (PRIN 2015 20152CB22L).

## Conflict of Interest Statement

The authors declare that the research was conducted in the absence of any commercial or financial relationships that could be construed as a potential conflict of interest.
