# Supplementary material for: The Impact of Mevastatin on HCV Replication and Autophagy of Non-Transformed HCV Replicon Hepatocytes Is Influenced by the Extracellular Lipid Uptake
Source: Front Pharmacol. 2019 Jun 26;10:718. doi: 10.3389/fphar.2019.00718 (PMC6611414; doi:10.3389/fphar.2019.00718)
Supplement: Supplementary file 1 [file DataSheet_1.pdf]

**SUPPLEMENTARY DATA**

**Supplementary Figure S1.**
**Analysis of cholesterol and autophagy levels in MMHD3 HCV Rep cells treated with Mevastatin and delipidated serum.**
(A) Representative images of filipin (left panels) and Bodipy 493/503 (right panels) staining of MMHD3 HCV-Rep cells untreated or treated with mevastatin as described in figure 1C (x400). Filipin and Bodipy 493/503 are specific fluorescent probes for cholesterol and neutral lipids, respectively. (B) MMHD3 HCV-Rep cells were infected with a retroviral vector encoding the RFP-LC3 reporter and left untreated or treated with mevastatin as described in figure 1C. Left panels: RFP-LC3; right panels: filipin (x650). Arrows indicate some of the LC3 vesicles that are positive to filipin staining. (C) MMHD3 HCV-Rep cells were treated with mevastatin for 3 days in the presence of normal or delipidated fetal bovine serum (FBS). Intracellular cholesterol content was visualized by filipin staining. Phase contrast (left panels) and filipin signal (right panels) are shown (x400). (D) MMHD3 HCV-Rep cells were infected with a retrovirus encoding the RFP-GFP LC3 reporter to measure the autophagic flux. Cells were cultured with delipidated fetal bovine serum and mevastatin for 3 days, or left untreated. Representative images of GFP, RFP and merge signals are shown (x800). (E) Intracellular cholesterol content was visualized in Beclin 1 silenced cells treated with mevastatin for 3 days in the presence of normal or delipidated FBS by filipin staining. Phase contrast (left panels) and filipin signal (right panels) are shown (x400).
