# Supplementary figures and images for: The Impact of Mevastatin on HCV Replication and Autophagy of Non-Transformed HCV Replicon Hepatocytes Is Influenced by the Extracellular Lipid Uptake
Source: Front Pharmacol. 2019 Jun 26;10:718. doi: 10.3389/fphar.2019.00718 (PMC6611414; doi:10.3389/fphar.2019.00718)

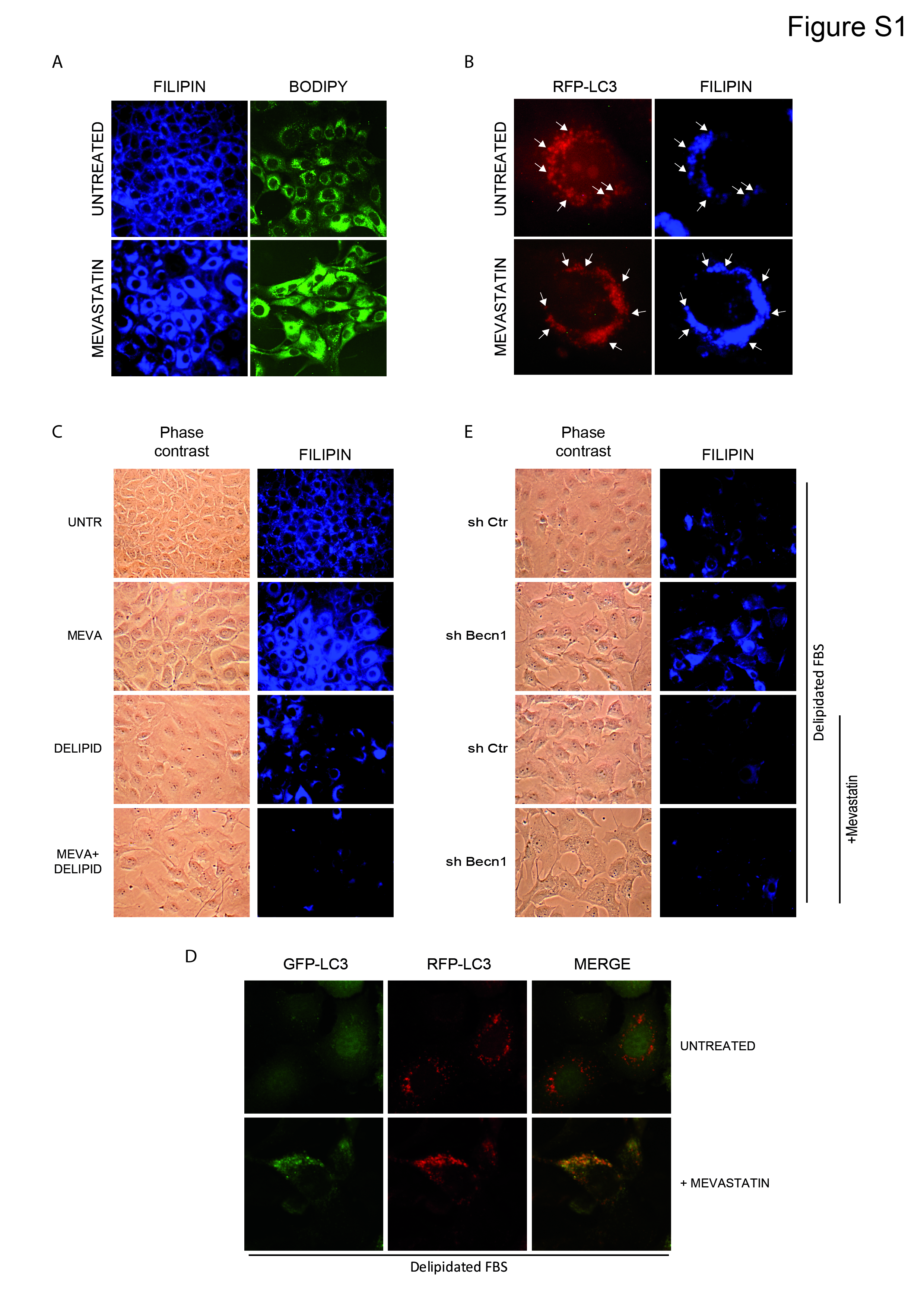

Supplement: Supplementary file 2 [file Image_1.tif]
